# *Notes from the Field:* Nontuberculous Mycobacteria Infections After Cosmetic Surgery Procedures in Mexico — United States, April–September 2025

**DOI:** 10.15585/mmwr.mm7541a2

**Published:** 2026-10-22

**Authors:** Neel Swamy, Michelle Sandoval-Rosario, Dumbani Kayira, Kiran M. Perkins, Rebecca Pierce, Ana C. Bardossy, Julie R. Gutman, Carolyn Herzig, Fernanda C. Lessa, Rhett J. Stoney, Julie Thwing, Paige Gable, Heather Moulton-Meissner, Tapati Mazumdar, Thomas Ewing, Mara Rauhauser, Seema Shah, Vladimir González, Miguel A. Nakamura, Yoshiyuki Acosta-Ramos, María E. Jiménez, Daniel C. Ehlman, Nirma D. Bustamante

**Affiliations:** ^1^Epidemic Intelligence Service, CDC; ^2^Division of Global Migration Health, National Center for Emerging and Zoonotic Infectious Diseases, CDC; ^3^Epidemiology and Immunization Services Branch, County of San Diego Health and Human Services Agency, San Diego, California; ^4^Division of Healthcare Quality Promotion, National Center for Emerging and Zoonotic Infectious Diseases, CDC; ^5^General Directorate of Epidemiology, Ministry of Health, Álvaro Obregón, Ciudad de México, Mexico.

**Summary**

**What is already known about this topic?**

Cosmetic surgery is a frequent reason that persons travel outside the United States for medical care (i.e., medical tourism) and has been associated with nontuberculous mycobacteria (NTM) infections.

**What is added by this report?**

At least five U.S residents developed NTM infections after facelift surgeries by one surgeon at one hospital in Jalisco, Mexico, during April–September 2025. Whole genome sequencing identified three closely related *Mycobacterium abscessus *isolates, suggesting a common infection source. Lapses in surgical instrument sterilization were identified during an inspection of the hospital, resulting in closure of the operating area and confiscation of the surgeon’s surgical instruments.

**What are the implications for public health practice?**

NTM infections are not nationally notifiable in the United States, making the detection of outbreaks associated with medical tourism difficult. Education about NTM risk after cosmetic surgery might expedite diagnosis and treatment of affected patients. Surveillance for adverse events associated with medical tourism could facilitate more timely detection and response to cosmetic procedure–associated NTM outbreaks.

## Investigation and Outcomes

Nontuberculous mycobacteria (NTM) infections, including those caused by *Mycobacterium abscessus*, are associated with outbreaks in health care settings, including those providing cosmetic surgery (*1*). NTM infections can be difficult to diagnose; routine bacterial cultures are often inadequate, and acid-fast bacilli cultures are typically needed. *M. abscessus* infections can be severe and affect multiple organ systems, even among immunocompetent persons (*2*,*3*). On December 22, 2025, the County of San Diego Health and Human Services Agency was notified of an *M. abscessus* infection in a resident who had traveled outside the United States for facelift surgery (i.e., a medical tourist) in Zapopan, Jalisco, Mexico, in September 2025 (patient A in California). Beginning eight days after surgery, from October to December 2025, patient A developed multiple seromas (i.e. fluid-filled pockets) on the face and neck, requiring antimicrobials and four drainage procedures. Patient A later received a diagnosis of *M. abscessus* infection.

Patient A reported communicating through social media with other U.S. residents who also had experienced infections after facelift surgeries performed by the same surgeon (surgeon X) at the same hospital (facility X). In February 2026, CDC and Mexico’s Ministry of Health launched a binational investigation to identify additional cases and potential infection sources. This activity was reviewed by CDC, deemed not research, and conducted consistent with applicable federal law and CDC policy.*

A confirmed case of cosmetic surgery–associated NTM was defined as laboratory evidence of a rapidly growing^†^ NTM in a patient who underwent cosmetic surgery by surgeon X in facility X during January 1, 2025–July 1, 2026. CDC coordinated with administrators of the social media network to encourage case reporting. A second California resident (patient B) and an Illinois resident (patient C) were part of this social media network and reported NTM infections after having had surgeries by surgeon X in July 2025. Whole genome sequencing (WGS) performed by CDC’s Clinical and Environmental Microbiology Branch confirmed the isolates from patients A, B, and C as *M. abscessus* sequence type (ST) 56 with one or two high-quality^§^ single nucleotide variant differences across a 99.64% sequenced core genome, indicating a likely common source. Through the social media network, CDC was notified of two additional persons who met the case definition: a Texas resident (patient D) and a Florida resident (patient E). Patient D had developed a *Mycobacterium fortuitum* infection after facelift surgery by surgeon X in April 2025. Patient E had developed an *M. abscessus* infection after facelift surgery by surgeon X in September 2025. Across the cluster, median interval from procedure date to symptom onset was 33 days (range = 4–112 days); median interval from procedure date to culture collection was 76 days (range = 47–116 days) (Figure). All patients sought treatment in the United States; no deaths occurred.  Some patients in the cluster are still receiving treatment (i.e., medications, corrective surgeries for scarring and disfigurement, and drainage procedures), whereas others have completed treatment. The isolate from patient E was confirmed as ST42 by WGS, and the isolate from patient D was a different species of NTM (*M. fortuitum*), suggesting these infections were not part of the same genetic cluster (ST56) as that of patients A, B, and C. This finding does not rule out the possibility of a common infection source in facility X or the health care environment (e.g., a water source).

**FIGURE:**
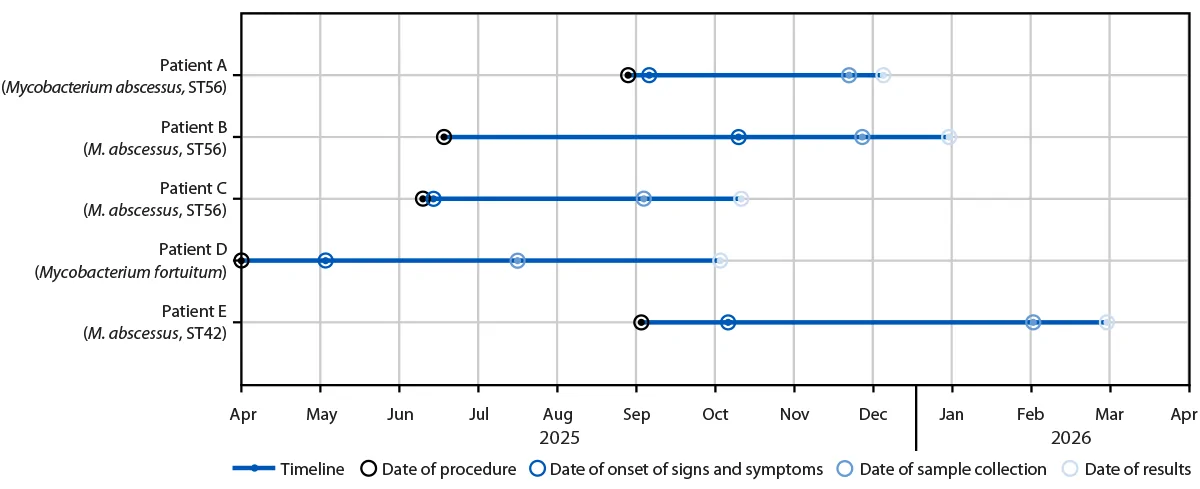
Timeline from procedure (April–September 2025) as well onset of signs and symptoms, sample collection for acid-fast bacilli culture, and culture result among U.S. residents who received cosmetic surgery in Mexico — United States, April 2025–March 2026 **Abbreviation:** ST = sequence type.

CDC and Mexico’s Ministry of Health continue to collaborate to identify additional cases and potential outbreak sources. During inspection of facility X in February 2026, lapses in surgical instrument sterilization were identified, resulting in closure of the operating area and confiscation of surgical instruments from surgeon X. CDC and the Mexico Ministry of Health have requested a list of U.S. residents who received care at facility X during April–September 2025, and the investigation is ongoing. As of October 19, 2026, five confirmed cosmetic surgery–associated NTM cases linked to surgeon X at facility X had been identified among U.S. residents across four U.S. states.

## Preliminary Conclusions and Actions

Providers should consider NTM infection among patients with postsurgical infections, especially after cosmetic procedures (*1*,*4*). Medical tourists traveling for medical care should be aware that facility accreditation and health care professional certification standards might vary outside the United States (*5*). CDC has developed the Medical Tourism Surveillance System (MTSS) to track adverse health outcomes linked to medical tourism. Although extrapulmonary NTM infections are not nationally notifiable, using MTSS could streamline reporting to CDC and help identify epidemiologically related infections that might indicate an outbreak. Improving awareness about infections associated with medical tourism, timely reporting to local health jurisdictions, and coordinated local, national, and international response, are central to protecting the health of U.S. travelers.

## Footnotes

* 45 C.F.R. part 46, 21 C.F.R. part 56; 42 U.S.C. Sect. 241(d); 5 U.S.C. Sect. 552a; 44 U.S.C. Sect. 3501 et seq.

† Rapidly growing NTM are defined by growth in culture within 7–10 days. Including this term in the case definition allows for grouping of NTM that are a common cause of postprocedural skin and soft tissue infections and that might have common environmental sources as part of an outbreak or cluster.

§ High-quality is the precision and confidence with which the sequencing tool can identify nucleotide differences. For this analysis, the minimum variant coverage was 10x, the minimum mean mapping quality was 30, and the single nucleotide variant relative abundance ratio was 0.75.
